# Comparison of Clinical Outcomes and Soft Tissue Laxity Between Robot-Assisted Functionally and Mechanically Aligned Total Knee Arthroplasty

**DOI:** 10.7759/cureus.91084

**Published:** 2025-08-27

**Authors:** Yohei Naito, Shine Tone, Gai Kobayashi, Masahiro Hasegawa

**Affiliations:** 1 Department of Orthopaedic Surgery, Mie University Graduate School of Medicine, Tsu, JPN

**Keywords:** clinical outcomes, functional alignment, laxity, rosa, total knee arthroplasty

## Abstract

Purpose

This study aimed to compare postoperative clinical outcomes and soft tissue laxity between functional alignment (FA) and mechanical alignment (MA) in robot-assisted total knee arthroplasty (TKA).

Methods

Thirty-one FA and 31 MA TKAs performed using a robotic system were included in this retrospective study. Range of motion (ROM), the 2011 Knee Society Score (2011 KSS), and the Forgotten Joint Score-12 (FJS-12) at 6 weeks, 3 months, and 6 months after TKA, respectively, were compared between the two groups. Intraoperative laxities were compared between the two groups.

Results

Although no significant differences were noted in postoperative ROM and the 2011 KSS, the FJS-12 scores at 3 and 6 months after TKA were significantly higher in the FA group (64.4 and 68.4) than those in the MA group (51.9 and 57.0) (p<0.05 and p<0.05). At 90° flexion, the medial laxity of 1.1 mm in the FA group was significantly lower than that of 2.3 mm in the MA group (p < 0.001).

Conclusion

FA TKA was superior to MA TKA in achieving better FJS-12 and greater medial stability at 90° flexion.

## Introduction

Mechanical alignment (MA) was introduced by Insall et al. in the 1980s and is widely used in total knee arthroplasty (TKA) [1]. Neutral alignment of the lower limb mechanical axis and proper femoral and tibial implant alignments have been accepted to achieve greater stability, lower rate of loosening, and higher clinical scores in TKA [2-5]. However, approximately 20% of patients are dissatisfied with TKA results [6]. Furthermore, recent studies have reported that postoperative lower limb alignment does not affect short- and long-term clinical outcomes [7-10]. Robot-assisted TKA has been proven to improve the accuracy of correcting lower limb alignment and component positioning and to reduce radiographic outliers [11, 12]. With this improved accuracy, functional alignment (FA) has recently been developed. FA TKA aims to restore joint line height, preserve native obliquity, and achieve balanced flexion-extension gaps with equal medio-lateral soft tissue tension by manipulating bone resections and fine-tuning implant positioning. Conceptually, FA TKA reduces the need for intraoperative periarticular soft tissue release while restoring native pre-arthritic knee kinematics [13].

ROSA (Zimmer Biomet, Warsaw, IN, USA) is an image-based robotic system, where the surgeon can proceed only if the landmark points show a correspondence with the preoperative radiographic planning; it is also available as an image-free option, relying solely on the correct quality of the acquisition for bone morphology and resections [14]. Cutting levels of the distal femur and proximal tibia, rotational alignment, and size of the femoral component can be determined using soft tissue laxity. Postoperative soft tissue can also be assessed.

To the best of our knowledge, no studies have compared postoperative patient-reported outcomes (PROMs) and soft tissue laxity between FA and MA TKAs using ROSA. The study questions were as follows: (1) Are there significant differences in PROMs between patients who underwent FA and MA TKAs? (2) Are there significant differences in soft tissue laxity between patients who underwent FA and MA TKAs? We hypothesized that PROMs in FA TKA would be better than those in MA TKA, and that better soft tissue laxity would be achieved in FA TKA than in MA TKA. The objectives of this study were to compare PROMs and soft tissue laxity between FA and MA TKAs using ROSA.

## Materials and methods

Study design

This study was approved by the Institutional Review Board of our Institution (Reference: H2018-083), and written informed consent was obtained from all patients. All the procedures were performed in accordance with the principles of the Declaration of Helsinki.

Thirty-one knees of 29 patients with varus osteoarthritis underwent MA TKA between February 2021 and December 2021, and 31 knees of 29 patients with varus osteoarthritis underwent FA TKA between October 2023 and August 2024. Overall, there were 11 male and 20 female participants with a mean age of 74.5 years (range, 56-84 years) and mean body mass index (BMI) of 26.8 kg/m2 (range, 18.4-38.8 kg/m2) in an FA group, and four male and 27 female participants with a mean age of 73.2 years (range, 59-87 years) and mean BMI of 26.9 kg/m2 (range, 20.9-44.6 kg/m2) in an MA group. The exclusion criteria of this study were valgus deformity, inflammatory arthritis, and prior knee surgery.

Surgical technique

A single surgeon with more than 30 years of experience performed the TKA procedures using the mid-vastus approach without patellar eversion. A similar type of medial congruent TKA design (Persona, Zimmer Biomet, Warsaw, IN, USA) was implanted using ROSA. After resection of the posterior cruciate ligament and removal of osteophytes, the deep medial collateral ligament (MCL) was released in the MA group, while the superficial MCL was left intact to maintain slight medial tightness. In the FA group, no soft-tissue release was performed. Thereafter, manual varus stress was applied throughout the range of motion (ROM) to tension the lateral collateral ligament, followed by manual valgus stress to tension the MCL. The maximum medial and lateral gaps (mm) were recorded at full extension and at 90° flexion.

Yee et al. reported excellent intra-observer reliability for extension medial (intraclass correlation coefficient (ICC) 0.967), good to excellent for extension lateral and flexion medial (0.928 and 0.905, respectively), and moderate to excellent for flexion lateral (0.885) during manual ligament tension assessment using an image-free robot-assisted TKA system (CORI, Smith & Nephew, Memphis, TN, USA) [15]. The cutting levels of the distal femur and proximal tibia were determined based on soft tissue laxity. After preoperative planning, the ROSA robotic system placed and held the cutting guide at the desired location, and the surgeon was able to cut the bone through the guide using a bone saw. In the MA group, distal femur and proximal tibia resections were performed perpendicular to the femoral and tibial mechanical axes.

In the FA group, distal femur and proximal tibia resections were performed to obtain a balanced extension gap; distal femoral resection was restricted to 0-1° varus, and proximal tibial resection to 0-2° varus. The distal femur resection angle was perpendicular to the femoral mechanical axis in thirty knees and 1° varus in 1 knee, and the tibia resection angle was 2° varus to the tibial mechanical axis in 29 knees and 1° varus in 2 knees in the FA group. Femoral flexion angle and tibial posterior slope were 4° in both groups. Following the distal femoral and proximal tibial cuts, a ligament tensor was applied with the knee at 0° extension and 90° flexion and tensioned to 30 lb. Medial and lateral extension and flexion gaps (mm) were recorded.

Femoral rotation and component size were determined to achieve similar extension and flexion gaps and to balance the medial and lateral gaps in flexion, while maintaining femoral external rotation ≥3° relative to the posterior condylar axis to avoid patellar maltracking in both groups. Rotational alignment of the tibial component was adjusted to the anteroposterior axis, connecting the attachment of the posterior cruciate ligament to the border of the medial third of the tibial tuberosity in both groups. Patellar resurfacing was performed in all cases, and all the components were fixed with cement. Following implantation of the femoral and tibial components, the angles of both components were validated using ROSA.

Evaluation of laxity

**Figure 1:**
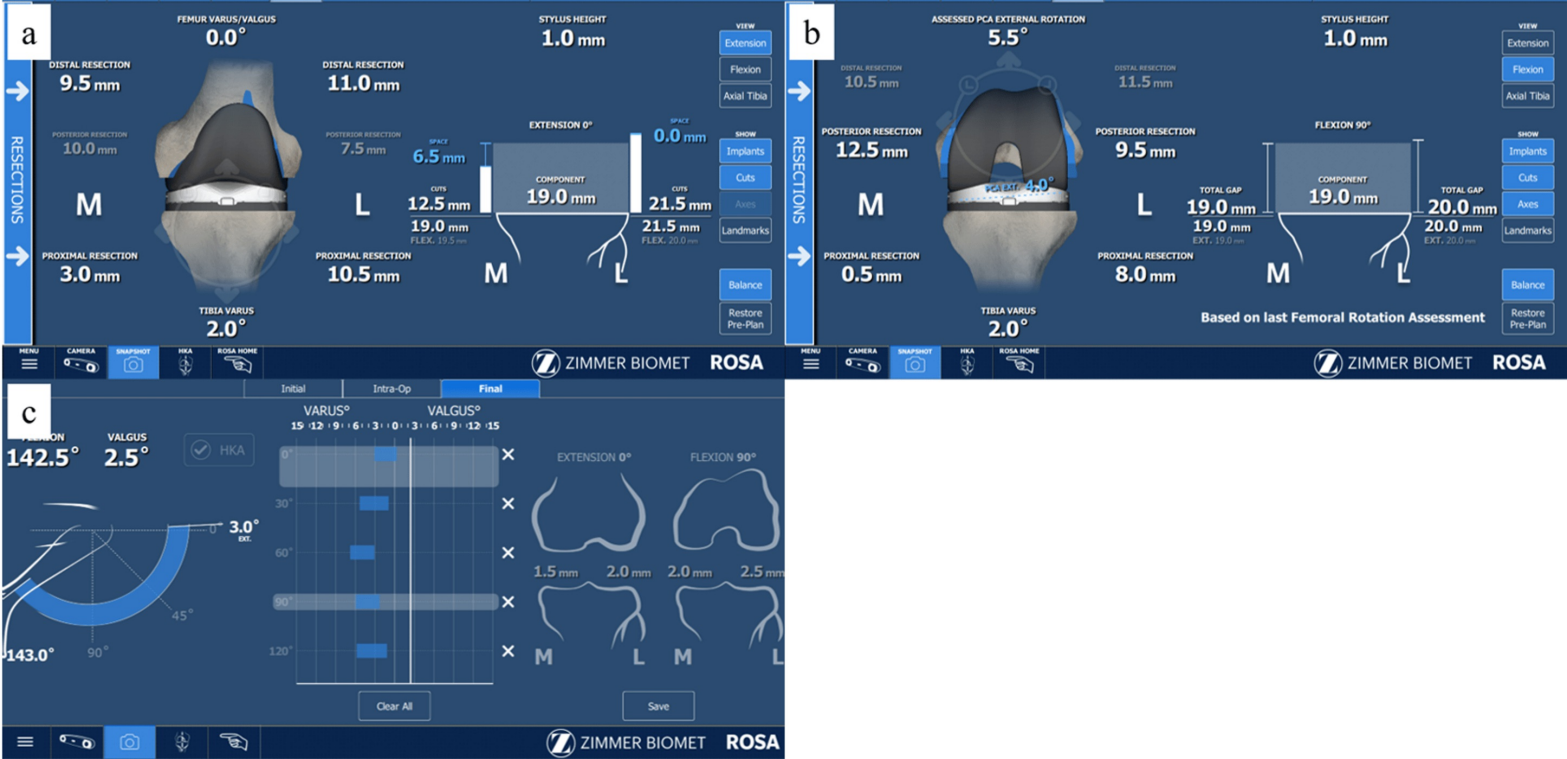
Measurements of medial and lateral laxities using ROSA Medial gap and lateral gap between the distal femoral and proximal tibial planned resection surfaces were displayed (a). Medial and lateral gaps between the posterior femoral planned and proximal tibial resection surfaces were displayed (b). Postoperative medial and lateral laxities were measured manually at each flexion angle (c). ROSA by Zimmer Biomet, Warsaw, IN, USA.

During planning at maximum extension, manual varus and valgus stresses were applied. The system displayed the medial and lateral gaps between the planned distal femoral and planned proximal tibial resection surfaces (Fig. 1a). Planned medial and lateral extension laxities at maximum extension were defined as these gaps minus 19 mm (implant thickness). During planning at 90° flexion, a ligament tensor was tensioned to 30 Ib. The system displayed the medial and lateral gaps between the planned posterior femoral and planned proximal tibial resection surfaces (Fig. 1b). Planned medial and lateral flexion laxities at 90° flexion were defined as these gaps minus 19 mm. After implantation of all the components and capsule closure, varus and valgus stresses were applied manually from 0° extension to full flexion, and medial and lateral laxities at each flexion angle were displayed on the monitor (Fig. 1c). Postoperative medial and lateral laxities at 0° extension and 90° flexion were recorded. Preoperative planned and postoperative medio-lateral laxity at each knee position was calculated by summing the medial and lateral laxities.

Clinical outcomes

Clinical evaluations were performed preoperatively at 6 weeks, 3 months, and 6 months postoperatively. The ROM and 2011 Knee Society Score (2011 KSS) [16] were assessed preoperatively and postoperatively. Forgotten Joint Score-12 (FJS-12) [17] was assessed postoperatively.

Radiographic assessment

**Figure 2:**
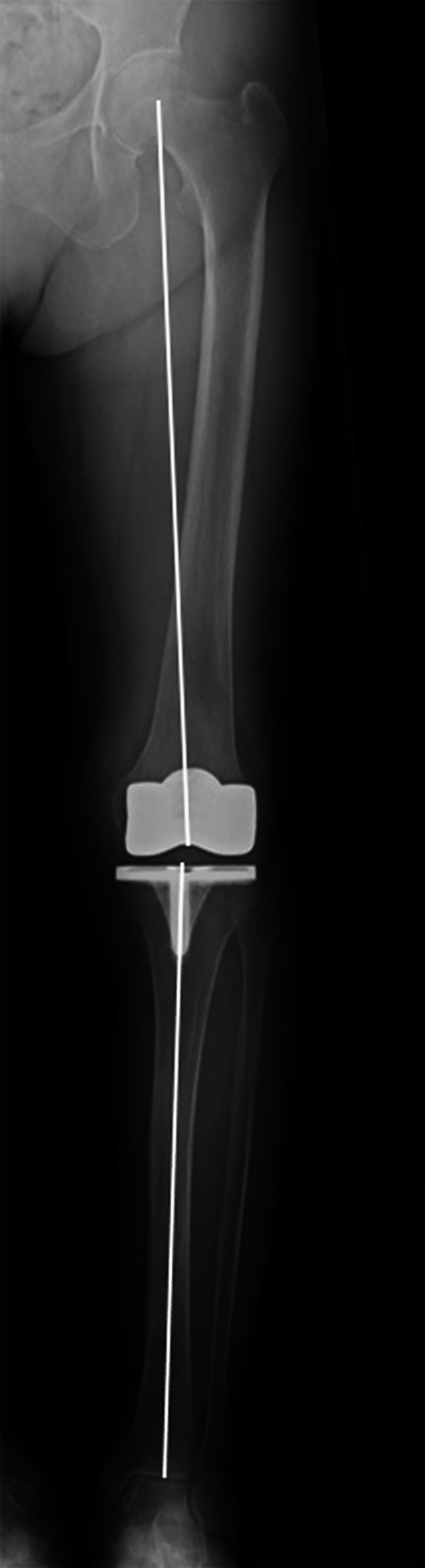
Postoperative long-leg anteroposterior weight-bearing radiograph in the functional alignment group.

Long-leg anteroposterior weight-bearing radiographs were obtained to measure the hip-knee-ankle angle (HKA) at 2 weeks postoperatively with the knees fully extended and the patellae facing forward (Fig. 2). Images were repeated when extension or patellar-forward alignment was inadequate. HKA was defined as the angle between the mechanical axes of the femur and tibia [18]. Valgus was denoted by positive values, whereas varus was denoted by negative values.

Statistical analysis

Power analysis was performed to determine sample size using the FJS-12 at 1-year post-TKA as the endpoint based on previous data: 52.9 for MA TKA and 73.9 for FA TKA [19]. Assuming that the true difference of the FJS-12 was 21.0 with a common standard deviation of 18.0, 12 cases were required in each group (α=0.05, 1-β=0.8). Differences between the MA and FA groups were compared using the Mann-Whitney U and chi-square tests. Because the statistical methods in this study differed from those in the previous report [19], we conducted a post hoc power analysis based on the observed between-group FJS-12 differences (two-sided α=0.05, n=31 per group). The Wilcoxon signed-rank test was used to compare continuous variables between the groups. Significance was determined using SPSS version 23 software (IBM Corp., Armonk, USA) (p < 0.05).

## Results

No significant differences were observed in demographic characteristics between the two groups (Table 1).

**Table 1:** Patient Demographics Sex, diagnosis, and surgical approach are reported as counts (n); all other variables are presented as mean ± standard deviation (SD). ^a^Chi-square test; ^b^Mann–Whitney U test FA, functional alignment; MA, mechanical alignment; ns, not significant

Variable	FA group	MA group	p-value
Sex (females/males)	11/20	4/27	ns^a^
Age (years)	74.6 ± 6.6	73.2 ± 7.1	ns^b^
Body mass index (kg/m^2^)	26.8 ± 4.7	26.9 ± 5.5	ns^b^
Diagnosis			
Osteoarthritis	31	31	ns^a^
Surgical approach			
Mid-vastus approach	31	31	ns^a^
Hip-knee-ankle angle (°)	−11.7 ± 4.3	−10.4 ± 3.4	ns^b^
Range of motion (°)	104.8 ± 20.5	112.4 ± 19.0	ns^b^
2011 Knee Society Score			
Overall	75.0 ± 21.2	81.8 ± 22.7	ns^b^
Symptoms	8.0 ± 5.5	8.2 ± 5.1	ns^b^
Satisfaction	13.2 ± 4.2	14.5 ± 5.9	ns^b^
Expectations	13.8 ± 1.2	13.4 ± 1.4	ns^b^
Functional activities	40.0 ± 15.7	45.7 ± 16.9	ns^b^

Clinical outcomes

The mean ROM at 6 weeks, 3 months, and 6 months postoperatively improved significantly compared to the mean preoperative ROM in both groups. There were no significant differences in the postoperative ROM between the two groups (Table 2). The mean overall 2011 KSS at 6 weeks, 3 months, and 6 months postoperatively were significantly greater than the preoperative 2011 KSS in both groups. There were no significant differences in the postoperative 2011 KSS between the two groups (Table 2). The mean FJS-12 was 50.2 (range, 14.6-90.6) at 6 weeks, 64.4 (range, 15-100) at 3 months, and 68.4 (range, 31.3-100) at 6 months postoperatively in the FA group. Mean FJS-12 was 45.5 (range, 4.2-79.2) at 6 weeks, 51.9 (range, 0-82.5) at 3 months, and 57.0 (range, 2.1-85.4) at 6 months postoperatively in the MA group. The FJS-12 in the FA group at 3 and 6 months postoperatively was significantly higher than that in the MA group (p < 0.05) (Table 2). 

**Table 2:** Postoperative clinical outcomes All values are presented as mean ± SD. ^a^Mann–Whitney U test FA, functional alignment; MA, mechanical alignment; ns, not significant

Variable	FA group	MA group	p-value
Range of motion			
6 weeks postoperatively (°)	119.8 ± 14.7	125.5 ± 14.0	ns^a^
3 months postoperatively (°)	124.8 ± 13.0	125.3 ± 13.4	ns^a^
6 months postoperatively (°)	126.5 ± 11.5	125.7 ± 16.1	ns^a^
2011 Knee Society Score			
Overall			
6 weeks postoperatively	106.2 ± 19.9	104.7 ± 23.0	ns^a^
3 months postoperatively	117.6 ± 22.6	113.4 ± 24.5	ns^a^
6 months postoperatively	127.7 ± 20.7	121.6 ± 27.9	ns^a^
Symptoms			
6 weeks postoperatively	18.4 ± 4.1	17.8 ± 4.3	ns^a^
3 months postoperatively	20.1 ± 3.4	19.4 ± 4.3	ns^a^
6 months postoperatively	21.0 ± 2.7	21.0 ± 3.5	ns^a^
Satisfaction			
6 weeks postoperatively	22.4 ± 6.7	21.9 ± 7.6	ns^a^
3 months postoperatively	25.5 ± 7.2	24.1 ± 6.6	ns^a^
6 months postoperatively	27.5 ± 6.5	26.0 ± 8.5	ns^a^
Expectations			
6 weeks postoperatively	9.8 ± 1.9	9.3 ± 1.7	ns^a^
3 months postoperatively	9.7 ± 2.2	9.4 ± 1.9	ns^a^
6 months postoperatively	9.9 ± 2.2	9.8 ± 2.5	ns^a^
Functional activities			
6 weeks postoperatively	55.6 ± 16.7	55.7 ± 15.4	ns^a^
3 months postoperatively	62.3 ± 17.8	60.6 ± 18.0	ns^a^
6 months postoperatively	69.3 ± 15.1	64.8 ± 19.0	ns^a^
Forgotten Joint Score-12			
6 weeks postoperatively	50.2 ± 21.3	45.5 ± 21.9	ns^a^
3 months postoperatively	64.4 ± 21.2	51.9 ± 19.5	< 0.05^a^
6 months postoperatively	68.4 ± 16.9	57.0 ± 22.6	< 0.05^a^

At 3 months postoperatively, the following FJS-12 items were significantly better in the FA group than in the MA group (lower scores indicate less joint awareness): in bed at night (1.0 ± 1.1 vs 1.8 ± 1.1, p < 0.01), sitting on a chair for more than 1 hour (1.0 ± 1.0 vs 1.6 ± 1.0, p < 0.05), walking for more than 15 minutes (1.0 ± 1.0 vs 1.7 ± 1.2, p < 0.05), taking a bath/shower (0.8 ± 0.7 vs 1.3 ± 0.8, p < 0.05), and traveling in a car (0.9 ± 1.0 vs 1.3 ± 0.9, p < 0.05). At 6 months postoperatively, the following items were significantly better in the FA group: in bed at night (0.8 ± 0.8 vs 1.5 ± 1.3, p < 0.05), sitting on a chair for more than 1 hour (0.8 ± 0.7 vs 1.4 ± 1.2, p < 0.05), and doing housework or gardening (1.5 ± 1.0 vs 2.0 ± 1.1, p < 0.05). A post hoc power analysis based on the observed between-group FJS-12 differences yielded power=0.68 at 3 months and power=0.61 at 6 months, indicating moderate power for the realized effects.

Laxity

Preoperatively, at maximum extension, planned medial laxity of the MA group was significantly lower than that of the FA group, and at 90° flexion, planned lateral laxity and medio-lateral laxity of the MA group were significantly greater than those of the FA group (Table 3). The mean planned femoral rotation angle was 3.6° (range, 3-5°) in the FA group and 3.9° (range, 3-6°) in the MA group, with no significant difference between the two groups.

**Table 3:** Preoperative planned medial and lateral laxities Data are presented as Mean ± Standard Deviation (SD). ^a^Mann–Whitney U test FA, functional alignment; MA, mechanical alignment; ns, not significant

Variable	FA group	MA group	p-value
Maximum extension			
Medial laxity (mm)	−0.9 ± 1.0	−1.9 ± 1.7	<0.005^a^
Lateral laxity (mm)	3.4 ± 1.8	2.9 ± 2.3	ns^a^
Medio-lateral laxity (mm)	2.4 ± 1.9	0.9 ± 3.2	ns^a^
90° flexion			
Medial laxity (mm)	1.1 ± 1.1	1.4 ± 2.3	ns^a^
Lateral laxity (mm)	1.2 ± 1.6	2.9 ± 2.4	<0.005^a^
Medio-lateral laxity (mm)	2.3 ± 2.4	4.3 ± 3.9	<0.05^a^

Postoperatively, at 0° extension, medial laxity was significantly lower, and lateral laxity was significantly greater in the MA group than in the FA group. At 90° flexion, medial laxity in the MA group was significantly greater than in the FA group. No significant differences were observed in postoperative medio-lateral laxity between the two groups at either 0° extension or 90° flexion (Table 4). In the FA group, no significant difference between medial and lateral laxity was found at 0° extension, whereas at 90° of flexion, medial laxity was significantly lower than lateral laxity. In the MA group, medial laxity was significantly lower than lateral laxity at 0° extension, while no significant difference was observed at 90° flexion.

**Table 4:** Postoperative medial and lateral laxities Data are presented as Mean ± Standard Deviation (SD). ^a^Mann–Whitney U test FA, functional alignment; MA, mechanical alignment; ns, not significant

Variable	FA group	MA group	p-value
Maximum extension			
Medial laxity (mm)	1.8 ± 1.0	1.2 ± 1.6	<0.05^a^
Lateral laxity (mm)	2.1 ± 1.3	3.0 ± 1.6	<0.05^a^
Medio-lateral laxity (mm)	3.9 ± 1.5	4.2 ± 1.8	ns^a^
90° flexion			
Medial laxity (mm)	1.1 ± 1.3	2.3 ± 1.6	<0.001^a^
Lateral laxity (mm)	3.8 ± 2.1	3.1 ± 2.3	ns^a^
Medio-lateral laxity (mm)	4.9 ± 2.7	5.4 ± 3.2	ns^a^

Radiographic findings

**Figure 3:**
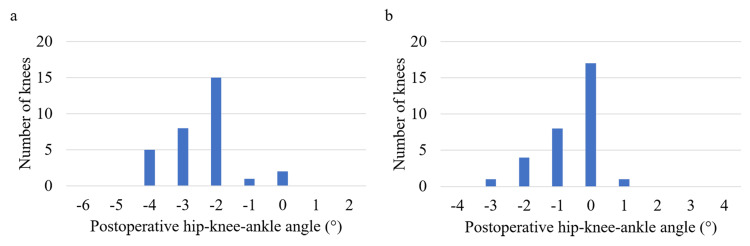
Distribution of the postoperative hip-knee-ankle angle (a) Functional alignment group; (b) Mechanical alignment group. Negative values indicate varus alignment, whereas positive values represent valgus alignment.

Mean postoperative HKA was −2.5° (range, −4-0°) in the FA group and −0.6° (range, −3-1°) in the MA group (p<0.001). No postoperative HKA outliers exceeding 3° from the target HKA were observed in either group (Fig. 3a, 3b). No knees exhibited loosening or mechanical failure.

## Discussion

The most important findings of this study were that the 3- and 6-month postoperative FJS-12 of patients who underwent FA TKA were greater than those of patients who underwent MA TKA, and that FA TKA was superior to MA TKA in achieving medial stability at 90° flexion.

Two studies reported that FA TKA performed with the assistance of the MAKO robotic system (Stryker, Mahwah, MI, USA) resulted in superior postoperative PROMs at >3 months and at 1-, 2-, and 5-year follow-ups compared to MA TKA performed using manual instruments [20, 21]. Parratte et al. found that at 6 months postoperatively, Knee Society Function Score (KSFS)in FA TKA using ROSA was significantly higher than that in adjusted MA TKA using a conventional technique [22]. However, it is not clear whether these differences were due to the use of the FA or robots. Jeffery et al. reported that FA TKA showed significantly better FJS-12, Oxford Knee Score (OKS), KSS, and KSFS at 1 year postoperatively than MA TKA, and both procedures were conducted with MAKO [23]. Lee et al. reported that the pain visual analog scale, Western Ontario and McMaster Universities Arthritis Index (WOMAC), FJS-12, and KSFS in FA TKA were significantly higher than those in MA TKA at 3 months, 6 months, or 1 year after MAKO-assisted TKA [19].

In this study, FJS-12 in the FA group was significantly higher than that in the MA group at 3 and 6 months after TKA using ROSA. In particular, at 3 months postoperatively, participants in the FA group reported less joint awareness than those in the MA group in bed at night, when sitting on a chair for more than 1 hour, when walking for more than 15 minutes, when taking a bath/shower, and when traveling in a car. At 6 months, the FA group reported less joint awareness in bed at night, when sitting on a chair for more than 1 hour, and when doing housework or gardening.

In this study, at 90° flexion, medial laxity was significantly greater in the MA group than in the FA group, even though the MA group had significantly lower medial laxity at 0° extension, consistent with the preoperative plan. Femoral external rotation did not differ significantly between the two groups. These findings suggest that medial soft tissue release can lead to increased medial laxity at 90° of flexion. Nakamura et al., in an in-vivo fluoroscopic analysis of posterior cruciate-sacrificing TKA, showed that a larger medial flexion gap leads to greater anterior femoral translation at complete condylar contact, whereas the lateral flexion gap has little effect [24]. Accordingly, the lower medial laxity at 90° flexion in the FA group may have prevented abnormal knee kinematics and contributed to the higher FJS-12.

Azukizawa et al. reported that medial component gaps at 90° flexion were negatively associated with the total 2011 KSS and 2011 KSS satisfaction subscale scores at 1 year after posterior stabilized (PS) TKA [25]. Hasegawa et al. also reported that medial laxity at 90° flexion was associated with patient dissatisfaction after PS TKA [26].

Parratte et al. revealed that no significant difference was observed between the medial and lateral gap laxities in extension, whereas a significant opening of the lateral gap was observed in flexion compared with extension in FA TKA using ROSA [22]. In this study, the FA group showed no significant difference between medial and lateral laxity at 0° extension, whereas lateral laxity was significantly greater than medial laxity at 90° flexion. Although a symmetric balance could not be obtained at 90° flexion in FA TKA in a previous study [22] and this study, lateral laxity in the native knee was reported to be significantly greater than medial laxity in both extension and flexion [27].

In this study, the planned medio-lateral laxity in the MA group was significantly greater than that in the FA group, whereas no significant difference was observed in the postoperative medio-lateral laxity between the two groups, and surgeons may need to be aware of this change. Meanwhile, Hasegawa et al. reported that medio-lateral laxity did not affect patient satisfaction or expectations in robot-assisted TKA [28].

No outliers exceeding 3° from the target HKA were observed in either group in this study. This finding was consistent with prior reports that ROSA achieved high accuracy in robot-assisted TKA, as assessed on long-leg anteroposterior weight-bearing radiographs, with no outliers exceeding 3° from the planned alignment [28, 29].

This study has some limitations. First, the FA TKA was performed within a restricted component alignment. The clinical outcomes in the patients with varus tibial component alignment >3° were reported to be poorer than those in the patients with neutral component alignment [4]. The mean HKA angle after FA TKA was reported to be −1.1° to −2.7° [18-22]. Similarly, the mean postoperative HKA angle in the FA group was −2.5° in this study. Second, laxity was measured manually, and the applied stress was not quantified. The effect of this could have been minimized by having a single experienced surgeon use a consistent technique throughout the study. Third, improvements in surgical technique over the two-year interval between the FA and MA groups might have influenced the postoperative results.

However, as all procedures were performed by a single surgeon with more than 30 years of experience in TKA, the impact of surgical technique differences on the outcomes is likely to be small. Fourth, this study was a retrospective and not a randomized controlled study. There might be inherent biases in patient selection, which could influence outcomes; however, the two groups were comparable at baseline with no significant differences in patient characteristics. Finally, the follow-up period was short; further studies are necessary to investigate the effects of FA TKA.

## Conclusions

We compared PROMs and soft tissue laxity between robot-assisted FA and MA TKA in varus knees. FA TKA demonstrated better FJS-12 at 3 and 6 months postoperatively and greater medial stability at 90° flexion compared to MA TKA. These findings suggest that FA TKA, by minimizing the need for soft tissue release, may contribute to superior early FJS-12.
